# Discovery and early validation of serum protein signatures in untreated multiple sclerosis patients: identification of candidate biomarkers for diagnosis and stratification

**DOI:** 10.3389/fimmu.2025.1579045

**Published:** 2025-08-21

**Authors:** Isabel Brichette-Mieg, Ana Alonso-Torres, Pablo Aliaga-Gaspar, José-Luis Rodríguez-Bada, Virginia Reyes-Garrido, Patricia Urbaneja-Romero, Carmen Muñoz, María Díaz-Sánchez, Elisa Martín-Montañez, María-del-Rosario Cabello-Porras, Begoña Oliver-Martos, Laura Leyva

**Affiliations:** ^1^ Neuroimmunology and Neuroinflammation Group, Biomedical Research Institute of Málaga-IBIMA Plataforma Bionand, Hospital Regional Universitario de Málaga, Málaga, Spain; ^2^ Cellular and Molecular Biology PhD Program, Faculty of Science, University of Malaga, Málaga, Spain; ^3^ Department of Neurology, Hospital Regional Universitario de Málaga, Málaga, Spain; ^4^ Biomedicine PhD Program, Faculty of Medicine, University of Malaga, Málaga, Spain; ^5^ Department of Neurology, Hospital Complejo Torrecárdenas, Almería, Spain; ^6^ Department of Neurology, Hospital Universitario Virgen del Rocío, Sevilla, Spain; ^7^ Department of Pharmacology, Faculty of Medicine, University of Malaga, Málaga, Spain; ^8^ Department of Cell Biology, Genetics and Physiology, Physiology Area, Faculty of Science University of Malaga, Málaga, Spain

**Keywords:** multiple sclerosis, serum biomarkers, proteomics, differentially abundant proteins, neuroinflammation, innate immunity, oxidative stress

## Abstract

**Background:**

Despite progress in serum biomarker research, reliable tools for early diagnosis and patient stratification in multiple sclerosis (MS) remain limited. This study uses proteomic profiling in untreated MS patients to identify early disease-associated biomarkers.

**Methods:**

We conducted an unbiased proteomic screen to capture broad serum protein expression profiles in a well-characterized discovery sample: 7 relapsing remitting MS (RRMS), 7 secondary progressive MS (SPMS), 4 with primary progressive MS (PPMS) alongside 6 healthy controls (HC). Twelve candidate biomarkers were subsequently validated by ELISA in an independent sample comprising 80 untreated MS patients (38 RRMS, 21 SPMS, 21 PPMS) and 21 age- and sex-matched HC from southern Spain.

**Results:**

In the discovery phase, 393 proteins were identified; 13 showed significant differences between MS patients and controls and 4 were dysregulated between PPMS and relapsing-onset MS (ROMS). These proteins were involved in immune responses, oxidative stress, and complement regulation. ELISA validation confirmed six differentially abundant proteins (DAPs) in MS patients compared to controls. Among these, BST1 levels were elevated in ROMS (*P*_adj_ = 0.0017), while FCGR3A showed significant increases in PPMS (*P*_adj_ = 0.034). PRDX6 levels were consistently elevated in both ROMS (*P*_adj_ = 0.044) and PPMS (*P*_adj_ = 0.001), as were APEH levels (ROMS vs. HC: *P*_adj_ = 0.038; PPMS vs. HC: *P*_adj_ = 0.009), both correlating with higher disability scores. In contrast, CFHR5 and MST1 levels were significantly reduced in ROMS (*P*_adj_ ≤ 0.001 for both). Besides, disease severity was significantly associated with higher MST1 and APEH levels. Functional enrichment analyses linked these proteins to *innate immunity, neuroinflammation*, and *metabolic regulation*.

**Conclusion:**

Our study identified six proteins involved in key pathological mechanisms such as inflammation, oxidative stress, immune regulation, and blood-brain barrier (BBB) integrity. Notably, the upregulation of PRDX6—linked to protein repair and neuroprotection in EAE models—may reflect a compensatory response to neuroinflammatory damage. Conversely, the downregulation of MST1, a molecule involved in immune signaling, could impair neuroprotective signaling and may drive neuroinflammation. These findings highlight PRDX6 and MST1 as particularly promising biomarkers for the diagnosis and monitoring of MS, meriting further validation in larger, longitudinal cohorts.

## 1 Introduction

Multiple sclerosis (MS) is a chronic autoimmune disorder characterized by demyelination, neurodegeneration, and inflammation of the central nervous system (CNS). It is the leading cause of non-traumatic neurological impairment in young adults worldwide. Although the precise cause of MS remains unclear, it is believed to result from a combination of genetic predisposition and well-defined environmental factors (1).

Diagnosing MS is challenging and typically requires evidence of lesions separated in time and space, which is most commonly obtained through magnetic resonance imaging (MRI) or clinical history, supported by paraclinical tests (2). The 2010 McDonald criteria and further revisions allow for earlier diagnosis in patients presenting with typical clinical syndromes (3, 4). However, despite improved sensitivity, misdiagnosis remains a significant concern, underscoring the need for emerging diagnostic biomarkers in body fluids to improve accuracy (5). Additionally, despite the availability of a wide range of therapies, a substantial percentage of patients continue to experience relapses and disease progression of the disease. Therefore, reliable prognostic biomarkers that can predict disease progression, severity, and treatment responses, and that are easily assessable in longitudinal studies or clinical trials, remain an unmet need in MS. While serial MRI scans have proven valuable in prospective studies, they lack the sensitivity to detect early events, where biomarkers assessing metabolic and biochemical changes in body fluids may precede the detection of myelin damage by MRI (6).

While promising biomarkers such as serum neurofilament light chain (NfL) and glial fibrillary acidic protein (GFAP) have shown utility in monitoring disease activity and severity, they lack specificity for early diagnosis and patient stratification (7, 8). Furthermore, blood levels of chitinase-3-like protein 1 (CHI3L1) have shown inconsistent results in distinguishing patients with MS (pwMS) from HC (9). Other promising biomarkers include osteopontin, C-X-C motif chemokine 13 (CXCL13), leptin, brain-derived neurotrophic factor (7), and soluble CD163 (10), although findings are sometimes conflicting.

Recent advancements in serum proteomics, including high-resolution mass spectrometry (HRMS) and proximity extension assays (e.g., Olink technology), have enabled large-scale and sensitive protein analysis, particularly in cerebrospinal fluid (CSF). However, results in plasma have been less consistent (11, 12) and validation in well-defined cohorts remains a significant challenge. Despite these technological advances, there remains a critical need for reliable biomarkers that can differentiate between MS phenotypes and predict disease progression. Current biomarkers often lack the sensitivity and specificity necessary for accurate diagnosis, especially in early disease stages (13), and are insufficiently sensitive to disease progression and severity. Moreover, many proposed biomarkers are not easily translatable to clinical practice due to methodological complexity or limited validation.

To address this gap, our study focuses on untreated MS patients to eliminate confounding effects of immunomodulatory therapies on protein expression. Furthermore, we compare protein profiles across distinct MS phenotypes—relapsing-remitting (RRMS), secondary progressive (SPMS), and primary progressive (PPMS)—to identify biomarkers associated with divergent disease mechanisms.

To achieve these goals, we conducted a cross-sectional, hypothesis-free proteomic study aimed at identifying novel serum biomarkers in MS. A stepwise, or “triangular” biomarker discovery strategy was employed. This approach began with an exploratory discovery phase using advanced proteomic techniques (ultra-high-performance liquid chromatography coupled with high-resolution mass spectrometry) to identify as many novel dysregulated serum proteins as possible in a small, well-characterized group of untreated patients. This approach, standard in exploratory proteomics, is designed to generate comprehensive, unbiased data to inform downstream analyses. Candidates emerging from this phase were subsequently reassessed in a validation phase using enzyme-linked immunosorbent assay (ELISA) in a larger independent sample.

## 2 Materials and methods

### 2.1 Ethics statement

The study was conducted at the Neurology Services of the following Spanish hospitals: Regional Universitario de Málaga (HRUM), Virgen del Rocío de Sevilla, and Torrecárdenas de Almería, in accordance with the principles of the Declaration of Helsinki, and was approved by our institutional ethics committees (Comité de Ética de la Investigación Provincial de Málaga; Comité Coordinador de Ética de la Investigación Biomédica de Andalucía). All study participants gave their written informed consent for inclusion.

### 2.2 Serum sample collection and storing

Peripheral blood samples for both the discovery and validation phase were collected during remission by venipuncture and allowed to clot spontaneously for 30 minutes. Serum was obtained by centrifugation following standard protocols of the HRUM-IBIMA Biobank, part of Andalusian Public Health System Biobank. The serum was aliquoted, immediately frozen at -80°C, and thawed only once prior to proteomic or ELISA analysis. Controls were selected from the HRUM-IBIMA Biobank, which is part of the Andalusian Public Health System Biobank. Control subjects were enrolled following standardized biobank protocols and were included in the study if they had no history of neurological, inflammatory, or autoimmune diseases and were not under any immunomodulatory treatment at the time of sampling (Healthy controls). Patients were recruited over a two-year period from the multiple sclerosis units of the neurology departments at the previously mentioned hospitals.

#### 2.2.1 Discovery sample

A total of 18 patients with clinically definite MS (CDMS) according to McDonald criteria (3, 4) (7 patients with RRMS, 7 with SPMS, and 4 with PPMS) were included in the discovery phase of the study as a screening sample, along with 6 subjects who served as healthy controls (HC). These patients were treatment-naïve or had undergone a washout period of at least 2.5 months between the change of immunomodulators. Patients with active infections or other comorbidities were excluded.

#### 2.2.2 Validation sample

Validation of the results was carried out in an independent sample of 80 untreated MS patients (38 with RRMS, 21 with SPMS and 21 with PPMS), all meeting the same inclusion and exclusion criteria as those in the discovery sample. Additionally, 21 age- and sex-matched HC were included. For statistical analysis, RRMS and SPMS patients were grouped as relapse-onset MS (ROMS). A *post-hoc* power analysis was conducted using the pwr package in R (version 4.5.0), considering a balanced one-way ANOVA model with the calculated Cohen’s f based on the protein abundance data. This analysis confirms that the validation sample was adequately powered to detect meaningful differences in protein abundance across the defined groups and revealed a statistical power of 0.86 to detect differences in protein abundance with a large effect size (Cohen’s f = 0.4) at a significance level of α = 0.05.

For both samples, demographic and clinical data were retrieved from medical records and are presented in **Tables 1** and **2**. The onset of the MS disease was defined as the first episode of focal neurological dysfunction indicative of MS for patients with RRMS and SPMS, or the onset of the progressive symptoms for those affected with PPMS. Disease duration was estimated as the number of years from the onset of the disease to the assessment of disability at the time of the sample collection. Disability was evaluated by means of the expanded disability status scale (EDSS) and progression of disability was assessed by the Multiple Sclerosis Severity Score (MSSS) (14), which relates clinical disability to disease duration. Active disease was defined according to the presence of bouts and/or lesions with gadolinium enhancement at MRI, at the time of inclusion in the study.

**Table 1:** Demographic and clinical characteristics of patients with multiple sclerosis (pwMS) and healthy controls (HC) in the discovery group, at time of sampling.

Characteristics	HC n=6	pwMS n=18			*P-*value
Age (years)	41.5 (30.0-53.5)	49.0 (41.0-54.2)			n.s.
Female, n (%)	4 (66.6%)	12 (66.6%)			n.s.
Characteristics		RRMS n=7	SPMS n=7	PPMS n=4	*P-*value
Age (years)		43 (30.0-53.5)	54 (49.0-55.0)	51.5 (43.5-55.75)	0.039
Female, n (%)		4 (57.1%)	4 (57.1%)	4 (100%)	n.s.
Disease duration: time from disease onset to blood collection (years)		2.5 (1.5-19.0)	14.0 (12.0-22.0)	2.5 (1.25-6.0)	0.028
EDSS score		1.5 (1.0-2.0)	5.5 (3.5-6.0)	3.75 (2.5-5.75)	0.002

Quantitative data are presented as medians with (interquartile ranges). *P*-values: Refers to *P*-values obtained from comparisons between healthy controls and MS patients using the Mann-Whitney test (age), and the chi-square test (gender). *P*-values for comparisons among the 3 clinical forms of MS were calculated using the Kruskal-Wallis test, followed by pairwise comparison tests (age, disease duration, and EDSS). RRMS, Relapsing Remitting MS; SPMS, Secondary Progressive MS; PPMS, Primary Progressive MS; EDSS, Expanded Disability Status Scale.

**Table 2:** Demographic and clinical characteristics of patients with multiple sclerosis (pwMS) and healthy controls (HC) in the validation group, at time of sampling.

Characteristics	HC N=21	pwMS N=80			*P-*value
Age (years)	44.0 (35.5-52.0)	43.50 (37.0-50.75)			n.s.
Female, n (%)	15 (71.4%)	59 (73.8%)			n.s.
Characteristics		RRMS n=38	SPMS n = 21	PPMS n = 21	*P-*value
Age (years)		39.5 (31.0-44.5)	45.0 (42.0-51.0)	50.0 (40.5-55.5)	< 0,001
Female, n (%)		30 (78.9%)	16 (76.2%)	13 (61.9%)	n.s.
Disease duration: time from disease onset to blood collection (years)		2.0 (0.87-13,5)	16 (10-21)	4.0 (2-9.5)	<0.001
EDSS		1.0 (1.0-1.5)	6.0 (4.0-6.0)	3.5 (2.75-6.0)	< 0.001
MDSS		1.77 (0.67-2.65)	5.61 (4.55-6.57)	7.27 (6.25-8.44)	1.5 x 10^-9^
Relapse Rate in previous year		1.0 (1.0-1.0)	0.0 (0-1.5)	0	<0.001
Number of participants with Gd T1 lesions		9/38	1/21	5/21	

Quantitative data are presented as median and (interquartile range). *p*-values: Refers to *p*-values obtained following comparisons between HC and MS patients by means of a Mann-Whitney test (age), and chi-square test (gender), as well as p values obtained following comparisons among the 3 clinical forms by means of a chi-square test (gender) or Kruskal-Wallis test followed by pairwise comparison tests (age, disease duration, EDSS, relapse rate). RRMS, Relapsing Remitting MS; SPMS, Secondary Progressive MS; PPMS, Primary Progressive MS; EDSS, Expanded Disability Status Scale.

### 2.3 Proteomic analysis in the discovery phase

Proteomic analysis in human serum was performed in the Proteomic Unit of the Research Support Central Services (SCAI) of the University of Málaga.

#### 2.3.1 Sample preparation and In-gel digestion and peptide extraction

The 12 most abundant proteins in serum samples were depleted using the Pierce™ Top 12 Abundant Protein Depletion Spin Columns kit (Life Technologies Europe, Bleiswijk, Netherlands). Samples were processed following the manufacturer’s instructions. The eluates from the columns were concentrated in a vacuum centrifuge to obtain 45 µL of depleted samples.

The samples were then trapped in a polyacrylamide matrix for gel-assisted proteolysis, where they were reduced with dithiothreitol, alkylated with iodoacetamide, and digested with trypsin (Promega, Madison, WI, USA). The resulting peptides were extracted with acetonitrile (ACN)/0.1% formic acid (FA) for 30 min at room temperature.

The samples were dried using a SpeedVac™ vacuum concentrator, re-dissolved in 50 µL of 0.1% FA aqueous solution, sonicated for 3 min, and centrifuged at 14,000 × g for 5 min. Subsequently, the samples were re-quantified in a NanoDrop™ (Thermo Fisher Scientific, Waltham, MA, USA), and 0.1% FA was added to standardize all samples to a protein concentration of 0.5 µg/µl before transfer to the injection vial.

#### 2.3.2 Ultra-high-performance liquid chromatography & high-resolution mass spectrometry

Samples were injected into an Easy nLC 1200 UHPLC system coupled to a hybrid linear trap quadrupole Orbitrap Q-Exactive HF-X mass spectrometer (ThermoFisher Scientific) and used software versions for data acquisition were Tune 2.9 and Xcalibur 4.1.31.9.

We used the same methodology as the one employed in a previous study (15) “HPLC solvents were as follows: solvent A consisted of 0.1% FA in water and solvent B consisted of 0.1% FA in 80% acetonitrile. Samples were then automatically loaded onto a trap column (Acclaim PepMap 100C18, 75 μm × 2 cm, 3 μm, 100 Å, ThermoFisher Scientific) at a flow rate of 20 μL/min and eluted onto a 50 cm analytical column (PepMap RSLC C18, 2 μm, 100 Å, 75 μm × 50 cm, Thermo Fisher Scientific). The peptides were eluted from the analytical column with a 120 min gradient ranging from 2% to 20% solvent B, followed by a 30 min gradient from 20% to 35% solvent B and finally, to 95% solvent B for 15 min before re-equilibration to 2% solvent B at a constant flow rate of 300 nL/min.

MS1 scans were performed from *m/z* 300 to 1750 at a resolution of 120,000. Using a data-dependent acquisition mode, the 20 most intense precursor ions of all precursor ions with +2 to +5 charge were isolated within a 1.2 *m/z* window and fragmented to obtain the corresponding MS/MS spectra. The fragment ions were generated in a higher energy collisional dissociation cell and detected in an Orbitrap mass analyzer at a resolution of 30,000. The dynamic exclusion time for the selected ions was 30 s. Maximal ion accumulation time allowed in MS and MS2 mode was 50 ms. Automatic gain control was used to prevent overfilling of the ion trap and was set to 3 × 10^6^ ions and 10^5^ ions for a full MS and MS2 scan, respectively.

The LTQ Velos ESI Positive Ion Calibration Solution (Pierce, IL, USA) was used for external calibration of the instrument prior to sample analysis. Internal calibration was performed using the polysiloxane ion signal from ambient air at m/z 445.120024.

To control for potential batch effects, all samples were analyzed in a single analytical run. Additionally, the injection order of the samples was randomized to minimize systematic analytical bias. Blank samples were injected between each sample to monitor instrument stability and detect potential carry-over.

#### 2.3.3 Data analysis for protein identification

Tandem MS (MS/MS) spectra were searched against the Homo sapiens SwissProt protein database canonical version using the SEQUEST^®^ HT search engine in Proteome Discoverer™ 2.2 (Thermo Fisher Scientific, Waltham, MA, USA). Search parameters included a precursor ion mass tolerance of 10 ppm, a fragment ion tolerance of 0.02 Da, and allowance for up to two missed tryptic cleavages. Peptide spectral matches (PSMs) and protein assignments were validated using the Percolator^®^ algorithm based on a target-decoy strategy, applying a strict false discovery rate (FDR) threshold of 1%. Identified peptides were grouped into proteins following the principle of parsimony, and only master proteins with at least two unique peptide sequences detected in all three biological replicates of any given experimental condition were retained for further analysis. As a quality control measure, a Thermo Scientific™ Pierce™ LC-MS grade bovine serum albumin (BSA) digestion standard was included throughout the workflow.

#### 2.3.4 Label-free relative quantification for differential expression analysis

Label-free quantitation was implemented using the Minora feature of Proteome Discoverer™ 2.2. (Thermo Fisher Scientific) The protein abundances were based on precursor intensities. Normalization was performed based on the “Total Peptide Amount” and the abundance ratios were calculated using the Protein Abundance Based approach. Hypothesis testing was carried out using an ANOVA based on the abundance of each of the individual proteins or peptides. Only proteins with ANOVA p<0.05 and higher abundance ratio (AR) than 2:1 or smaller than 1:2 for patients: controls were considered as significantly dysregulated.

To represent the data graphically we used a normalized value corresponding to the log_2_ fold change (FC) in protein abundance ratio between samples, that is, a 1-point difference in the log_2_ FC equates to a 2× higher abundance ratio of protein.

The network of interactions between the differentially enriched proteins was constructed by using the STRING tool (https://sting-db.org/; v.12.0, accession date 11 Nov 2024). Additionally, pathway enrichment analysis was carried out with Reactome (https://reactome.org/PathwayBrowser; v. 90, accession date 01 Dec 2024).

### 2.4 Enzyme immunoassays in the validation phase

To validate the proteomic findings, twelve of the dysregulated proteins in the discovery phase were assessed for further evaluation in the validation group, due to limited serum availability. The immunoassays were performed in serum samples by commercial enzyme linked immunosorbent assay (ELISA) kits: (Actinin-alpha 1 [ACTN1], N-Acylaminoacyl-Peptide Hydrolase [APEH], Bone Marrow Stromal Cell Antigen 1 [BST1], Complement Factor H-Related 2 [CFHR2], Complement Factor H-Related 5 [CFHR5], Elastase-Neutrophil Expressed [ELANE], Peroxiredoxin-6 [PRDX6], Brain phosphoglycerate Mutase 1 [PGAM1], S100 calcium-binding protein A6 [S100A6] and Fc fragment of IgG low affinity IIIa receptor [FCGR3A] (all of them from Antibodies-online, Aachen, Germany); Macrophage Stimulating 1-Hepatocyte Growth Factor Like [MST1] (from Boster Biological Technology, Pleasanton CA, USA) and Human Proprotein Convertase 9 [PCSK9] (from USA R&D Systems, Minneapolis, USA). Serum samples were measured in duplicate and were diluted according to manufacturers’ procedures, when needed. Details regarding the sensitivity, specificity, lot numbers, and coefficients of variation of the commercial ELISA kits used in this study are provided in **Supplementary Table 1**.

### 2.5 Statistical analysis in the validation phase

Normality of the data was evaluated using the Shapiro-Wilk test, but most variables followed a non-parametric distribution. Protein concentrations between MS patients and HC were analyzed using the Mann-Whitney test. Subsequently, protein levels across different subject groups (HC, ROMS, and PPMS) were initially compared using the Kruskal-Wallis (KW) test. When the KW test indicated significant differences, pairwise comparisons were performed using a matrix pairwise comparison, with statistical significance set at a Bonferroni-adjusted alpha threshold of 0.05 (*P*_adj_).

Data are presented as median and interquartile ranges (IQR) for quantitative variables, and as percentages for qualitative variables. All statistical analyses were conducted on the original data, with logarithmic transformation applied solely to enhance the graphical representation of PRDX6 protein enrichment distribution across groups.

Effect size was estimated using Cliff’s delta, a non-parametric statistic suitable for ordinal or non-normally distributed data and unequal sample sizes. Median fold changes (FC) were reported to reflect group differences. Interpretation of Cliff’s delta followed the thresholds proposed by (16), with values of |δ| between 0.11 and 0.33 considered small, 0.33 to 0.47 medium, and ≥0.47 large. All effect size calculations were performed using the effsize package in R (version 4.5.0).

Protein associations to clinical variables such as disease duration, annual relapse ratio, EDSS at inclusion, or MSSS at inclusion were analyzed using Spearman’s rank correlation followed by a multivariable linear regression model, adjusting for sex and age at sampling. Results were considered significant at a *P*-value <0.05.

Statistical analyses were performed using the SPSS software (version 28.0.1.1). Graphical representations were generated using SPPS or Reactome PA library (version 1.5) in R (version 4.3.3).

## 3 Results

### 3.1 Discovery phase by UHPLC-HRMS

#### 3.1.1 Descriptive characteristics of the discovery sample

The demographic and clinical characteristics of the study participants are summarized in **Table 1**. No significant differences in age at sampling or sex were observed between pwMS and HC. Among pwMS, RRMS patients were younger than those with SPMS or PPMS. As anticipated, SPMS patients exhibited longer disease duration and higher EDSS scores compared to RRMS and PPMS patients.

#### 3.1.2 Proteomic analysis in human serum

In this study, a total of 1432 proteins were identified, of which 393 met the criteria of being “Master” proteins within their protein group, complying with a strict cut-off of 1% false discovery rate (FDR) and being identified by, at least, two unique peptide sequences.

**Figure 1:**
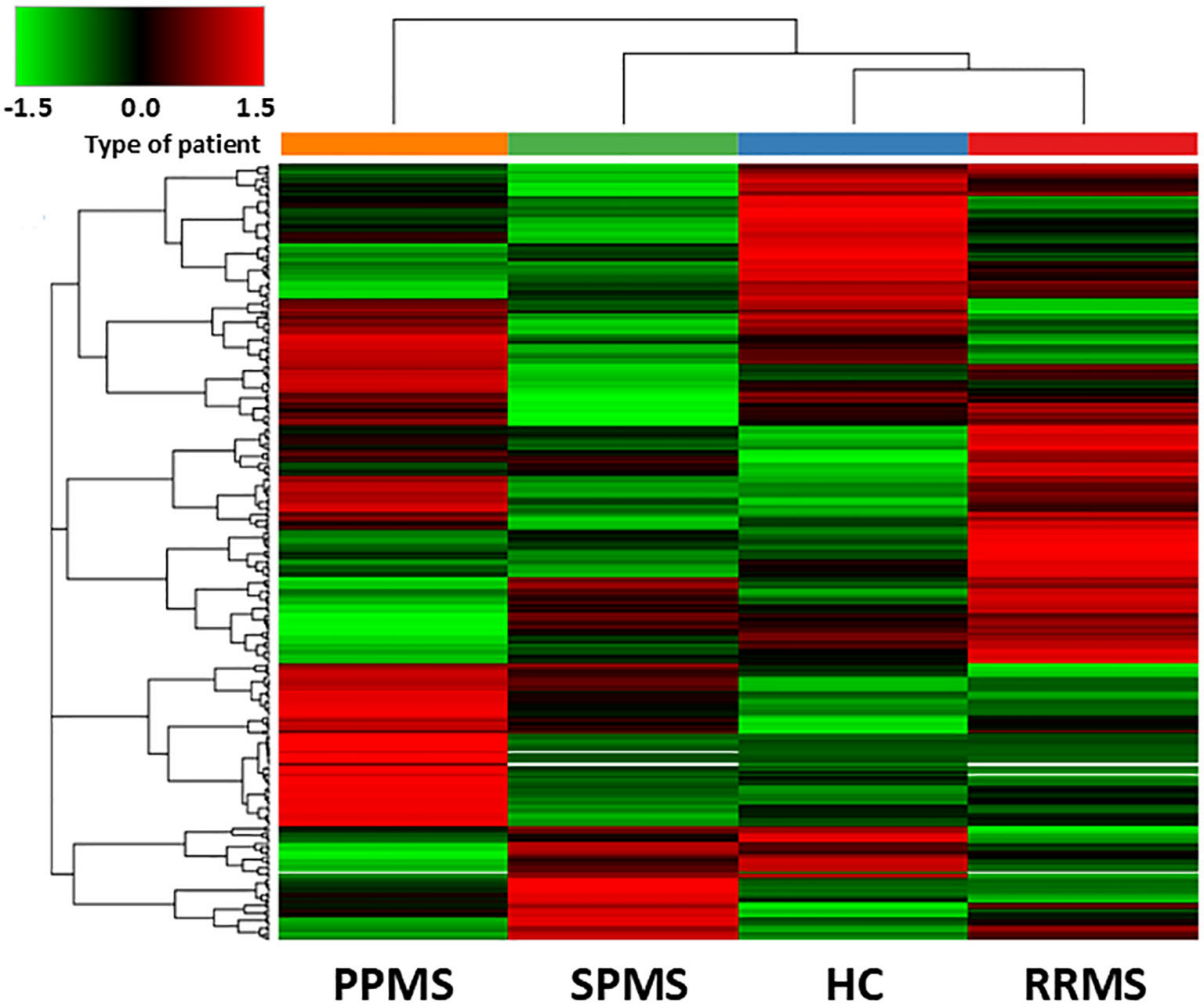
Hierarchical clustering of differentially abundant proteins in the discovery sample. The heatmap shows the hierarchical clustering of the 393 differentially expressed proteins in Controls and MS patients fulfilling the following criteria: being master proteins with at least two unique peptide sequences; being present in the 3 biological replicates of any of the conditions selected; showing a cut-off of 1% false dicovery rate. Relative abundance levels are shown on the green to red scale, with the numbers indicating the fold difference from the overall mean. The red colour of the tile indicates high abundance, green indicates low abundance and black indicates null values. The clustering of the control and MS groups is represented by the dendrogram at the top (PPMS in orange, SPMS in green, RRMS in red and controls in blue). The clustering of individual serum proteins is represented by the dendrogram on the left.

Every MS clinical form showed a unique protein profile, perfectly distinguishable from controls and from the other MS clinical forms, as depicted in the heat map of **Figure 1**.

**Figure 2:**
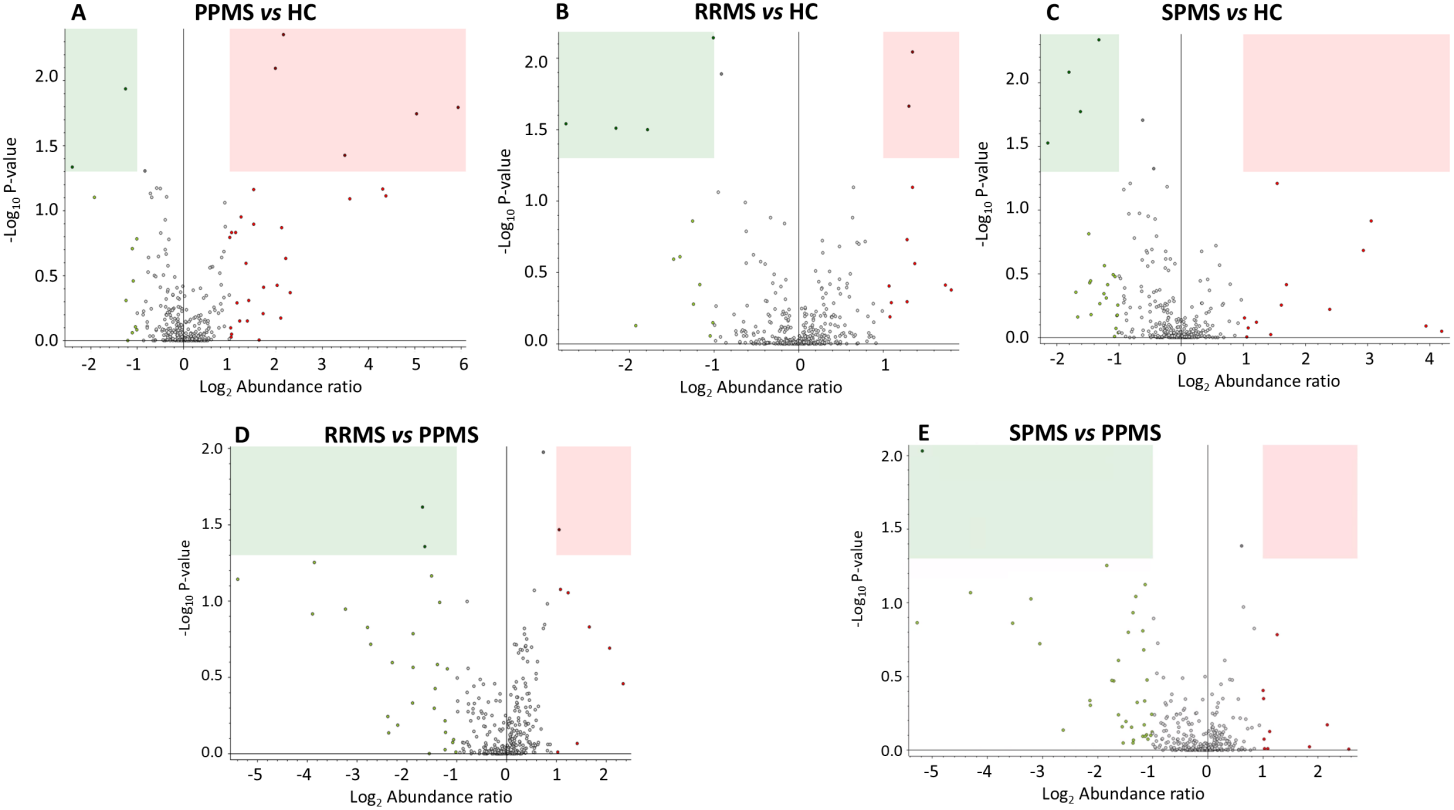
Volcano plots representing label-free protein quantification, illustrating differences in protein abundance across multiple comparisons in the discovery sample: **(A)** PPMS patients *vs.* HC, **(B)** RRMS patients *vs.* HC, **(C)** SPMS patients *vs.* HC, **(D)** RRMS patients *vs*. PPMS patients, and **(E)** SPMS patients *vs.* PPMS patients. The x-axis represents the log_2_-fold change (FC) in protein abundance ratio. Values greater than 1 indicate proteins with an abundance ratio at least 2-fold higher in the first group of the comparison, while values less than -1 indicate proteins with an abundance ratio at least 2-fold higher in the second group. The y-axis shows the negative log_10_ of the p-value, with higher values indicating greater statistical significance. Proteins with an abundance ratio (AR) FC less than 0.5 and *P*-value < 0.05 (decreased AR) are highlighted within green rectangles, while those with an AR FC greater than 2 and and *P*-value < 0.05 (increased AR) are shown within pink rectangles.

**Figure 2** illustrates the significantly dysregulated proteins across the different clinical forms of MS (RRMS, PPMS, and SPMS) and with HC. In the comparison of DAP between the various clinical forms of MS and controls, seven proteins were upregulated and six were downregulated. In PPMS patients, five proteins (S100A6, ELANE, PRDX6, PGAM1 and BST1) were significantly elevated, while two proteins (CFHR5 and FCGR3A) were significantly reduced compared to HC. Additionally, seven dysregulated proteins were identified in RRMS or SPMS patients relative to HC. Among these, MST1 and CFHR2 were enriched in RRMS patients, whereas isoform 2 of A1BG, IGKC, immunoglobulin kappa light chain (IGKLc) and FCGR3A exhibited a lower abundance ratio (AR) in RRMS patients compared to HC. Similarly, FCGR3A, IGKC, IGKLc and ACTN1 also displayed reduced AR in SPMS patients relative to controls, as detailed in **Table 3**.

**Table 3:** Proteins dysregulated in primary progressive (PPMS), relapsing-remitting (RRMS) and secondary progressive multiple sclerosis (SPMS) patients compared to controls (HC).

Accession	Gene	Protein description [OS=Homo sapiens]	PPMS vs. HC		RRMS vs HC		SPMS vs. HC	
			AR	*P-*value	AR	*P*-value	AR	*P*-value
P06703	S100A6	Protein S100-A6	61.323	0.016				
P08246	ELANE	Neutrophil elastase	32.889	0.018				
P30041	PRDX6	Peroxiredoxin-6	11.284	0.037				
P18669	PGAM1	Phosphoglycerate mutase 1	4.490	0.004				
Q10588	BST1	ADP-ribosyl cyclase/cyclic ADP-ribose hydrolase 2	3.966	0.008				
Q9BXR6	CFHR5	Complement factor H-related protein 5	0.422	0.011				
P08637	FCGR3A	Low affinity immunoglobulin gamma Fc region receptor III-A	0.190	0.046	0.224	0.031	0.327	0.016
P26927	MST1	Hepatocyte growth factor-like protein			2.547	0.009		
P36980-1	CFHR2	Complement factor H-related protein 2			2.470	0.021		
P04217-2	A1BG	Isoform 2 of Alpha-1B-glycoprotein			0.498	0.007		
P01834	IGKC	Immunoglobulin kappa constant			0.290	0.031	0.287	0.008
P0DOX7		Immunoglobulin kappa light chain			0.149	0.028	0.227	0.029
P12814-1	ACTN1	Alpha-actinin-1					0.401	0.004

AR, abundance ratio.

Proteins that showed significant differences across all MS clinical forms, along with their AR, are summarized in **Table 4**. Notably, PGAM1 and APEH were enriched by at least threefold in PPMS compared to RRMS patients, whereas PCSK9 exhibited a lower AR in PPMS than in the RRMS group. In contrast, only one protein, ELANE, was significantly enriched in PPMS compared to SPMS patients, as illustrated in **Table 4** and **Figure 2**.

**Table 4:** Proteins dysregulated in primary progressive (PPMS) compared to relapsing-remitting (RRMS) or secondary progressive multiple sclerosis (SPMS) patients.

Accession	Gene	Protein description [OS=Homo sapiens]	PPMS vs. RRMS		PPMS vs. SPMS	
			AR	*P*-value	AR	*P*-value
P08246	ELANE	Neutrophil elastase			36.127	0.009
P18669	PGAM1	Phosphoglycerate mutase 1	3.209	0.024		
P13798	APEH	Acylamino-acid-releasing enzyme	3.103	0.044		
Q8NBP7-1	PCSK9	Proprotein convertase subtilisin/kexin type 9	0.481	0.034		

AR, abundance ratio.

**Figure 3:**
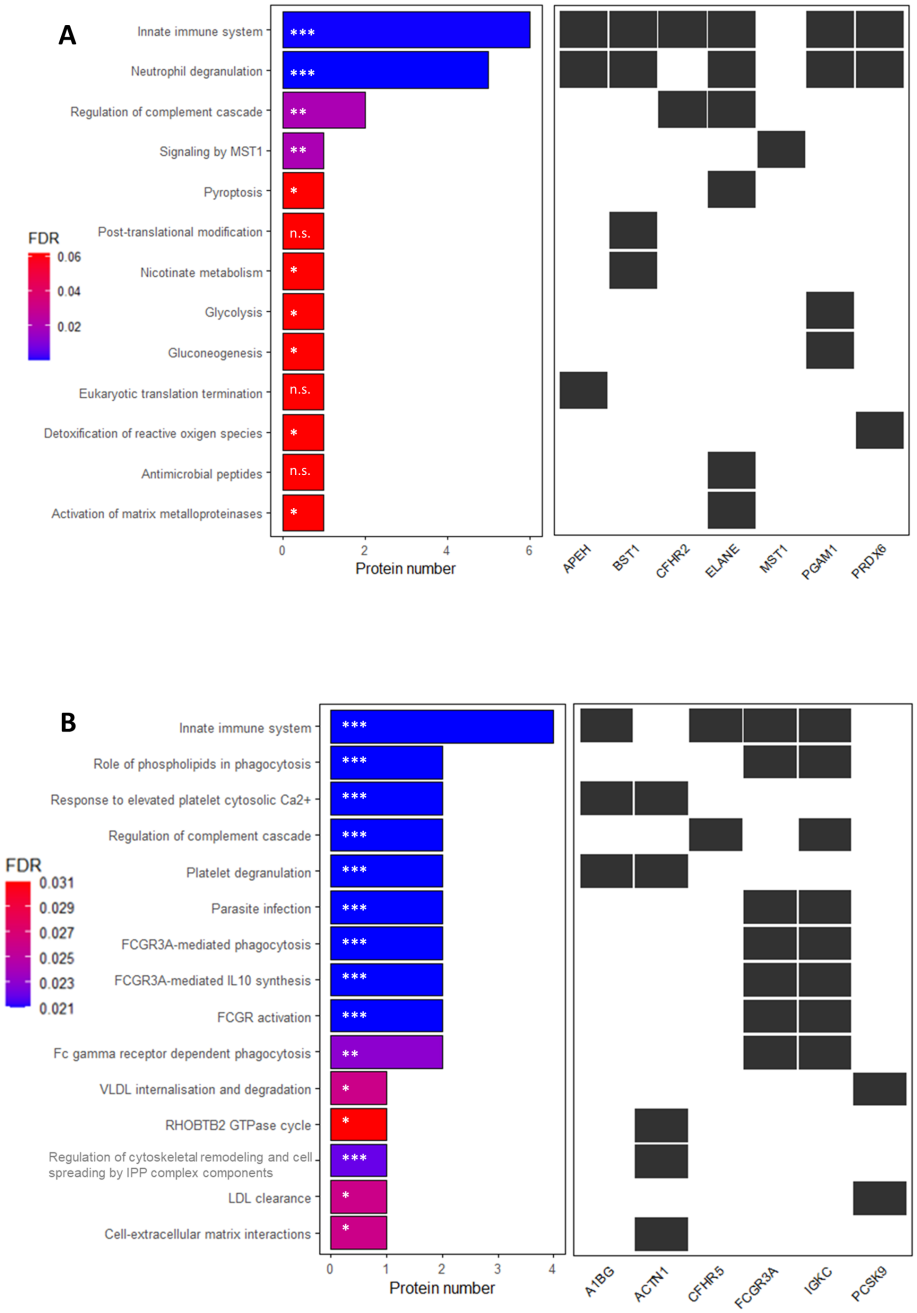
Reactome pathway enrichment analysis of serum proteins with significantly altered expression in patients with multiple sclerosis compared to controls in the discovery sample. X-axis shows the number of proteins up- **(A)** or downregulated **(B)** in the comparison. **(A)**: * *P*-value ≤ 0.03; ** *P*-value = 0.003; ***P-value= 5.4 x 10^-6^; n.s.= non-significant; **(B)**: *P-value ≤ 0.015; ** *P*-value < 0.01; *** *P*-value ≤ 0.005. The *P*-value is corrected for false discovery rate (FDR) using the Benjamini-Hochberg method. FDR-adjusted P-values are indicated in a colour scale on the left.

We mapped these proteins to Reactome pathways; however, neither S100A6 nor IGKLc could be assigned to any entity. To gain further insights into the biological pathways associated with the DAPs, we separately analyzed upregulated and downregulated proteins in pwMS vs. HC. Most upregulated DAPs mapped to the *Innate Immune System* and *Neutrophil Degranulation* pathways, while most downregulated DAPs also mapped to the *Innate Immune System*, as shown in **Figure 3**.

Next, we performed a comprehensive analysis of the signaling pathways associated with DAPs in each group comparison, as detailed in **Table 5**. Most proteins dysregulated between PPMS and HC were involved in the *Innate Immune System* pathway, while those altered in RRMS or SPMS compared to HC were primarily linked to *FCGR3 activation*. Additionally, DAPs between PPMS and RRMS were associated with *VLDLR internalization and degradation*, whereas those between PPMS and SPMS were involved in *Pyroptosis*.

**Table 5:** Mapping of differentially abundant proteins to reactome pathways in patients with different clinical forms of multiple sclerosis compared to controls or among clinical forms.

Group comparisons	Proteins	Reactome pathway	FDR	*P*-value
PPMS vs. HC	ELANE, PRDX6, PGAM1, BST1, CFHR5, FCGR3A	*Innate Immune System*	3.06 e^-4^	6.9 e^-6^
	ELANE, PRDX6, BST1, PGAM1	*Neutrophil degranulation*	0.002	8.4 e^-5^
	ELANE, CFHR5	*Regulation of complement cascade*	0.025	0.003
RRMS vs. HC	FCGR3A, IGKC	*FCGR3 activation*	0.012	0.001
		*Role of phospholipids in phagocytosis*	0.012	0.002
		*FCGR3A-mediated IL-10 synthesis*	0.012	0.002
	CFHR2, IGKC	*Regulation of complement cascade*	0.012	0.003
	A1BG, FCGR3A, CFHR2, IGKC	*Innate Immune System*	0.012	0.003
	MST1	*Signaling by MST1*	0.012	0.003
SPMS vs. HC	FCGR3A, IGKC	*FCGR3 activation*	0.006	4.3 e^-4^
		*Role of phospholipids in phagocytosis*	0.006	5.5 e^-4^
		*FCGR3A-mediated IL-10 synthesis*	0.006	6.9 e^-4^
		*Leishmania phagocytosis*	0.006	9.3 e^-4^
		*Fc gamma receptor (FCGR) dependent phagocytosis*	0.006	0.001
	ACTN1	*Regulation of cytoskeletal remodeling and cell* sp*reading by PINCH-ILK-Parvin complex components*	0.011	0.003
PPMS vs. RRMS	PCSK9	*VLDLR internalisation and degradation*	0.026	0.004
		*LDL clearance*	0.026	0.005
		*Plasma lipoprotein assembly, remodeling, and clearance*	0.027	0.019
		*Post-translational protein phosphorylation*	0.027	0.027
		*Regulation of Insulin-like Growth Factor transport and uptake by IGFBPs*	0.031	0.031
	APEH, PGAM1	*Neutrophil degranulation*	0.026	0.005
		*Innate Immune System*	0.029	0.029
	PGAM1	*Gluconeogenesis*	0.026	0.007
		*Glycolysis*	0.027	0.020
		*Glucose metabolism*	0.027	0.023
	APEH	*Eukaryotic Translation Termination*	0.027	0.027
PPMS vs. SPMS	ELANE	*Pyroptosis*	0.012	0.002
		*Activation of matrix metalloproteinases*	0.012	0.003
		*Regulation of complement cascade*	0.012	0.011

RRMS, Relapsing Remitting MS; SPMS, Secondary Progressive MS; PPMS, Primary Progressive MS.

#### 3.1.3 Functional enrichment analysis based on STRING database

**Figure 4:**
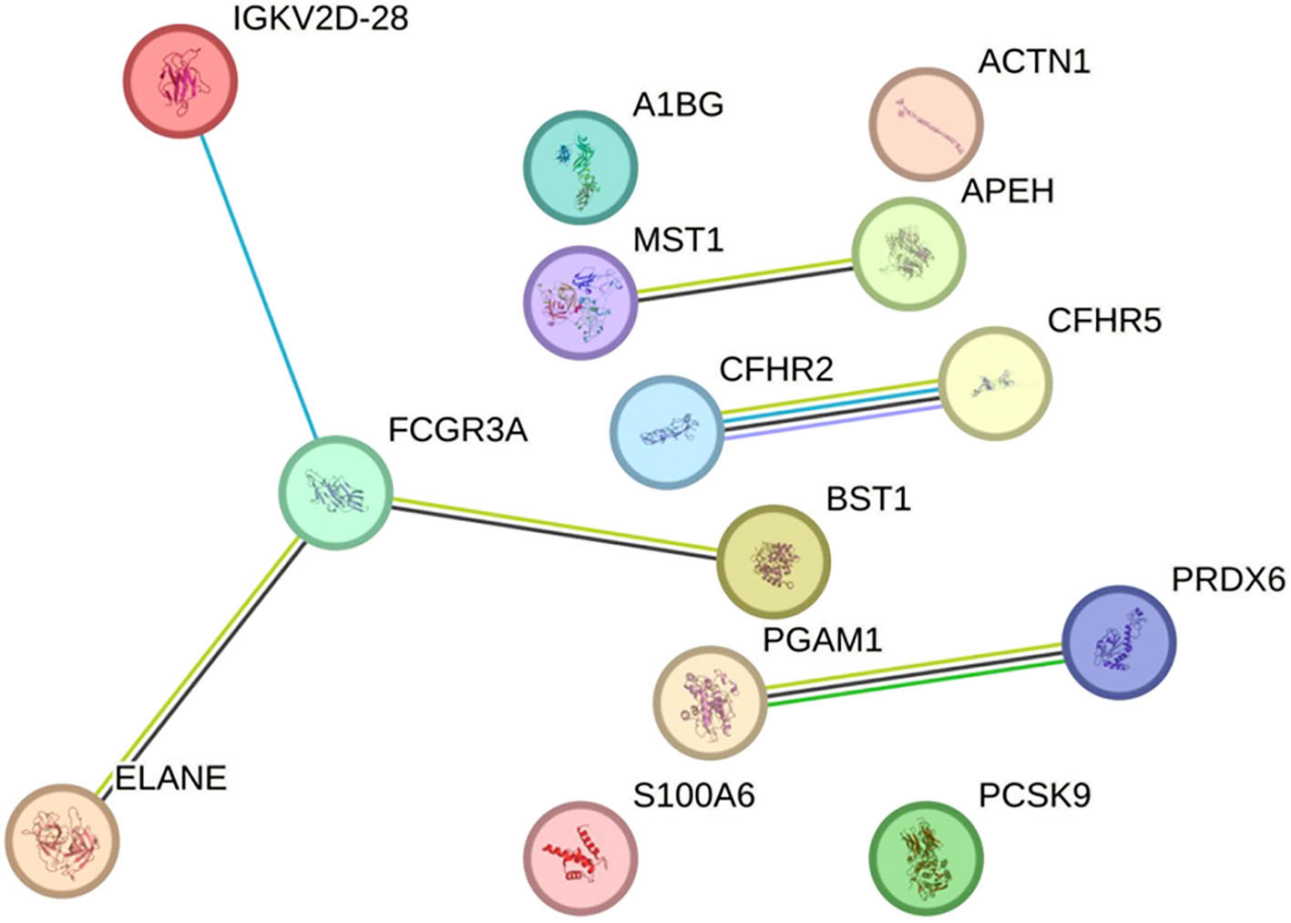
Protein-protein interaction network from the STRING database in the discovery sample. The network represents the interactions of differentially abundant proteins identified in the discovery phase. Each node corresponds to a protein, and connecting lines denote interactions between them. Interaction types are depicted using color-coded edges: deep sky blue for known interactions (database-sourced), green for predicted interactions (gene neighbourhood), yellowish green for text-mining evidence, black for co-expression, and light purple for protein homology. The network comprises 14 nodes, 6 edges, and an average local clustering coefficient of 0.643. Protein-protein interaction enrichment analysis yielded a *P*-value of 0.00078, indicating a statistically significant enrichment. This result suggests that the proteins are biologically connected as a functional group. An interactive version of the figure is available at the following link: https://version-12-0.string-db.org/cgi/network?networkId=b2fnseh1skSc.

The 15 identified DAPs in pwMS were analyzed using the STRING tool. While IGKC and IGKLc did not have entries in this database, the variable region of the light chain (IGKV2D-28) did, and was therefore included in the analysis. The resulting protein-protein interaction networks are depicted in **Figure 4**, showing 14 protein nodes and their interactions. The figure highlights four distinct networks: one comprising four nodes (IGKV2D-28, FCGR3A, ELANE, and BST1) with three interactions, and three smaller networks, each with two connected proteins (PGAM1-PRDX6, CFHR2-CFHR5, and MST1-APEH). The remaining proteins are represented as isolated nodes.

### 3.2 Validation phase by enzymoimmunoassays

#### 3.2.1 Descriptive characteristics of the validation sample

The descriptive characteristics of validation group are displayed in **Table 2**. The demographic characteristics of the validation sample at sampling did not differ significantly between MS patients and controls, in terms of sex and age. Patients with RRMS were, on average, younger than those with SPMS (*P =* 0.002) and PPMS (*P <* 0.001), showing no statistical differences between the two progressive forms. The male: female ratio did not differ significantly between MS participants in the 3 clinical forms. Disease duration was higher in the SPMS group than in RRMS and PPMS patients (*P <* 0.001, for both comparisons). EDSS score, as expected, was significantly lower in RRMS than in both progressive forms (*P <* 0.001, for both). MSSS score was also significantly lower in RRMS than in the progressive forms (*P <* 0.00005 when compared to SPMS patients and *P <* 1 x 10^-7^ when compared to PPMS patients). This score was also significantly lower in SPMS patients when compared to PPMS patients (*P* = 0.002). Active disease by Gadolinium-enhancing T1 lesions was detected in 9 patients of the RRMS group, in 1 from SPMS and in 5 from PPMS.

#### 3.2.2 Dysregulated proteins in MS patients *versus* HC and between PPMS and ROMS patients

In the discovery phase of our study, we identified 13 dysregulated proteins in MS patients compared to HC, with 7 proteins exhibiting higher AR and 6 showing lower ARs in pwMS. Notably, two of these proteins (ELANE and PGAM1), along with two additional ones (APEH and PCSK9), were dysregulated between PPMS and ROMS.

During the replication phase, we selected 12 of the 15 dysregulated proteins for ELISA validation, constrained by limited serum availability and by the fact that multiple studies have reported alterations in immunoglobulin levels (e.g., alpha, gamma, kappa, and lambda) in blood and CSF from MS patients, but with high inconsistency in results across studies.

In the validation phase, six proteins exhibited differential expression when analyzing MS patients as a whole, grouping all clinical forms together, compared to HC. Notably, BST1, PRDX6, and APEH were significantly elevated in MS patients, with *P*-values of 0.002, 0.002, and 0.004, respectively. Receiver operating characteristic (ROC) curves for these proteins in the classification of MS and HC showed the following areas under the curve (AUC) where the predictive state was MS [BST1 (AUC= 0.733, CI= 0.625-0.840); PRDX6 (AUC= 0.720, CI= 0.599-0.841); APEH (AUC= 0.712, CI= 0.599-0.826)]. Conversely, CFHR5, MST1 and CFHR2 levels were reduced in pwMS compared to HC, with *P*-values of 0.001, 0.006, and 0.04, respectively. The AUC for these proteins, where the predictive state was HC, was [CFHR5 (AUC= 0.792, CI= 0.655-0.929); MST1 (AUC=0.700, CI= 0.552-0.848); and CFHR2 (AUC= 0.650, CI= 0.528-0.772)].

**Figure 5:**
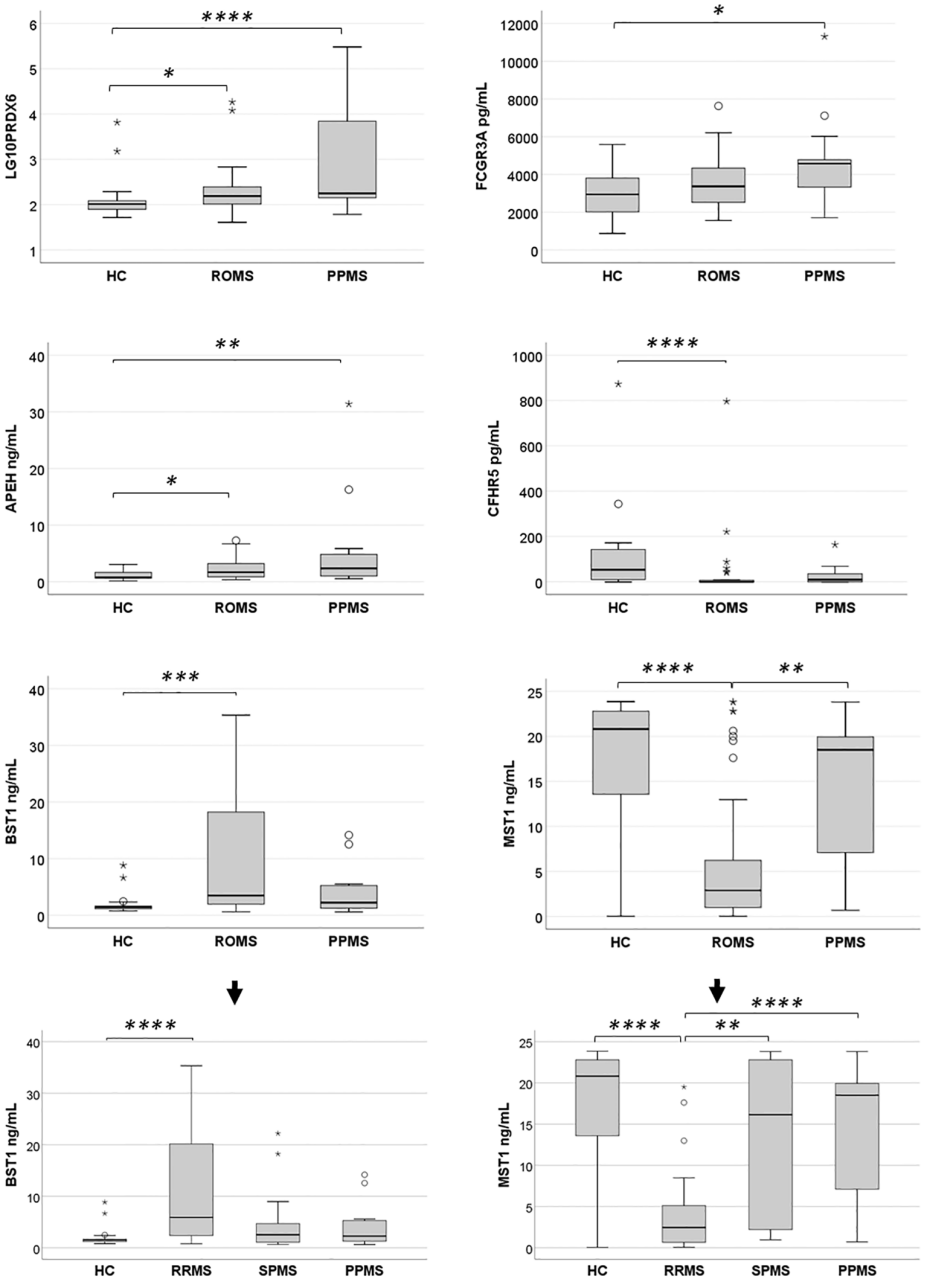
Expression levels of six differentially abundant proteins in healthy controls (HC) and untreated multiple sclerosis (MS) patients in the validation sample. Protein expression levels were evaluated in a validation sample comprising HC, relapsing-onset MS (ROMS), and primary progressive MS (PPMS) groups. Initial comparisons across groups were performed using the Kruskal-Wallis test. When significant differences were observed, pairwise comparisons were conducted with a Bonferroni-adjusted alpha threshold (*P*_adj_ = 0.05). The results are presented as box plots: horizontal bars represent the median, while the box edges denote the 25^th^–75^th^ percentiles. Whiskers extend to the 10^th^ and 90^th^ percentiles. Statistical analyses were conducted on untransformed data, with logarithmic transformation applied only to improve visualization of PRDX6 protein enrichment across groups. Further stratification of BST1 and MST1 levels among the three MS clinical forms and HC revealed that the observed differences in the ROMS group were primarily driven by the RRMS subgroup. *P_adj_<0.05; **P_adj_<0.01: ***P_adj_<0.005; ****P_adj_≤0.001.

When MS patients were stratified by clinical form (ROMS or PPMS), six proteins exhibited differential expression compared to HC, with slight variations from the findings observed when analyzing MS patients as a whole (**Figure 5**). Given the non-normal distribution of these proteins, data are presented as median and interquartile ranges in **Table 6**. The Kruskal-Wallis test showed a differential enrichment of PRDX6 (*P*-value*=* 0.002), FCGR3A (*P*-value *=* 0.039), APEH (*P*-value *=* 0.009), BST1 (*P*-value *=* 0.002), CFHR5 (*P*-value *=* 0.002) and MST1 (*P*-value *=* 0.0002) across the groups. Additionally, CFHR2 exhibited a trend toward lower expression, particularly in PPMS patients (*P*-value *=* 0.066).

**Table 6:** Serum levels of the 6 dysregulated proteins in the validation sample.

Proteins	Statistic	HC	RRMS	SPMS	PPMS
PRDX6 (pg/ml)	Median	103.07	158.75	141.84	177.64
	IQR	74.78 - 123.95	93.07 - 253.18	110.87 - 220.77	137.84 - 8960.00
FCGR3A (pg/ml)	Median	2951.5	3078.6	3934.1	4576.4
	IQR	2017.4 - 3814.7	2375.3 - 4158.5	3319.0 - 5026.9	2961.3 - 4822.6
APEH (ng/ml)	Median	0.79	1.46	2.02	2.4
	IQR	0.65 - 1.80	0.79 - 3.17	1.52 - 3.80	0.92 - 4.86
BST1 (ng/ml)	Median	1.50	5.89	2.53	2.27
	IQR	1.18 - 1.68	2.30 - 20.28	1.07 - 5.27	1.26 - 5.32
CFHR5 (pg/ml)	Median	53.86	0	0	10.28
	IQR	10.17 - 157.73	0.0 - 27.42	0.0 - 4.15	0.0 - 42.95
MST1 (ng/ml)	Median	20.81	2.45	16.14	18.50
	IQR	8.08 - 22.78	0.64 - 5.20	2.09 - 22.78	6.88 - 20.13

HC, healthy controls; RRMS, Relapsing-Remitting MS; SPMS, Secondary Progressive MS; PPMS, Primary Progressive MS; IQR, interquartile range.

Pairwise comparisons revealed that PRDX6 levels were significantly elevated in both PPMS and ROMS patients compared to HC, with Bonferroni adjusted *P*_adj_ of 0.001 and 0.044, respectively, consistent with findings from the discovery phase. The median fold increases were 1.72 for PPMS and 1.51 for ROMS. Effect size analysis indicated a large effect for PPMS vs. controls (Cliff’s δ = 0.62) and a medium effect for ROMS vs. controls (Cliff’s δ = 0.37). No significant difference was observed between PPMS and ROMS patients (FC = 1.14; Cliff’s δ = 0.29).

Unlike the discovery phase, ELISA validation showed a significant enrichment of FCGR3A in PPMS patients compared to HC (*P*_adj_ = 0.034; FC = 1.55; Cliff’s δ = 0.49), indicating a large effect size.

For APEH, although elevated levels were observed only in PPMS vs. RRMS in the discovery sample, validation in a larger sample revealed significant increases in both PPMS (*P*_adj_ = 0.009; FC=3.04) and ROMS (*P*_adj_ = 0.038; FC = 2.19) relative to HC. Effect size analysis showed a large effect for PPMS vs. HC (Cliff’s δ = 0.50) and a medium effect for ROMS (Cliff’s δ = 0.39). No other statistically significant differences were observed among groups.

In the discovery phase, BST1 levels were elevated across all MS subtypes, though significance was reached only in PPMS. However, ELISA validation revealed a significant increase in BST1 levels exclusively in ROMS patients compared to HC (*P*_adj_ = 0.0017), with a FC of 2.35 and a large effect size (Cliff’s δ = 0.51). Further stratification showed that this effect was primarily driven by the RRMS subgroup (*P*_adj_ = 0.001, FC = 3.94 and Cliff’s δ = 0.66).

Conversely, CFHR5 was significantly reduced in ROMS vs. HC during validation (*P*_adj_ = 0.001), with a FC of 0.018 and a large negative effect size (Cliff’s δ = –0.64). In the validation phase, a decrease in CFHR5 expression was also observed in PPMS patients, although this difference did not reach statistical significance.

While MST1 had been identified as enriched in RRMS compared to HC in the discovery group, ELISA validation showed an opposite pattern, with significantly lower levels in ROMS compared to both HC (*P*_adj_ = 0.001) and PPMS (*P*_adj_ = 0.007). Further analysis revealed that this reduction was driven by markedly low MST1 levels in RRMS patients. Specifically, RRMS patients had significantly reduced MST1 levels relative to HC (*P*_adj_ < 0.0001; FC = 0.12; Cliff’s δ = −0.62), SPMS (*P*_adj_ = 0.006; FC = 0.15; Cliff’s δ = −0.56), and PPMS (*P*_adj_ = 0.001; FC = 0.13; Cliff’s δ = −0.75), with large effect sizes in all comparisons.

The other proteins dysregulated in PPMS patients in the proteomic analysis when comparing to HC (S100A6, ELANE, PGAM1), RRMS (PGAM1 and PCSK9) or SPMS patients (ELANE) failed to show differential enrichment in the sera by ELISAs.

Discrepancies observed between the discovery and validation phases may reflect differences in sample size, analytical platform sensitivity, or sample composition. These findings underscore the importance of complementary methodologies and subgroup analyses to accurately validate and interpret biomarker relevance across heterogeneous MS populations.

In this study, several protein pairs demonstrated mild but statistically significant correlations in their expression levels, including FCGR3A and CFHR2 (Spearman’s ρ = -0.369, *P =* 0.001), BST1 and MST1 (ρ = -0.417, *P <* 0.0008), BST1 and PRDX6 (ρ = 0.384, *P <* 0.0002), and APEH and PRDX6 (ρ = 0.555, *P =* 1 × 10^-8^).

#### 3.2.3 Mapping of differentially abundant proteins to Reactome pathways

**Figure 6:**
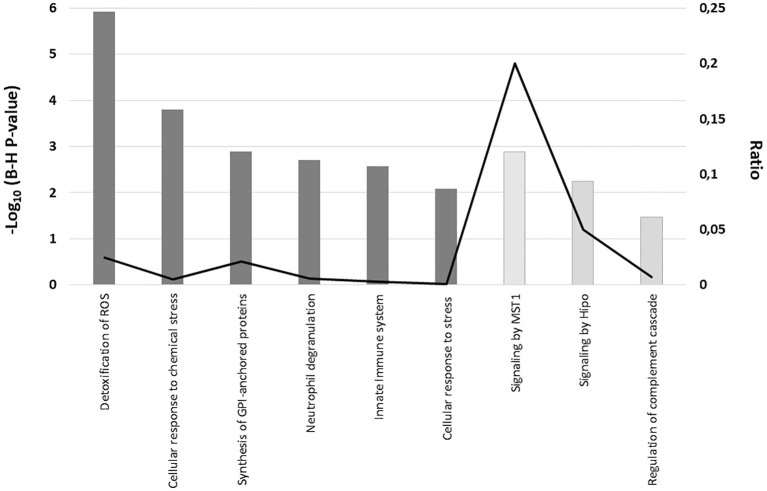
Reactome pathway enrichment analysis of serum proteins with significantly altered expression in MS patients compared to controls in the validation sample. The graph displays the cellular pathways enriched in the proteomic profiles of the upregulated proteins (dark grey) and downregulated proteins (light grey) in serum from MS patients as compared with healthy controls after correcting for multiple testing (Benjamini–Hochberg p-value), left axis). The black line represents the ratio of differentially expressed proteins in our validation sample within a specific pathway to the total number of proteins involved in that pathway, as defined by the reference dataset (right axis).

In the validation sample, network analysis using Reactome indicates that proteins upregulated in MS patients show enrichment in pathways related to Cellular response to stress [e.g., cellular response to chemical stress, detoxification of reactive oxygen species (ROS)], the Immune system [e.g., innate immune response, neutrophil degranulation], and Metabolism of proteins [e.g., synthesis of GPI-anchored proteins]. In contrast, proteins downregulated in MS samples are enriched in Signal transduction pathways [e.g., signaling by MST1, signaling by Hippo], as well as in Immune system pathways [e.g., regulation of the complement cascade], as shown in **Figure 6**.

#### 3.2.4 Associations with clinical characteristics

Among pwMS, the EDSS at sample collection time exhibited mild but significant correlations with FCGR3A (Spearman’s ρ = 0.303, *P =* 0.013), BST1 (ρ = -0.325, *P =* 0.005), PRDX6 (ρ = 0.246, *P =* 0.034), and MST1 (ρ = 0.522, *P =* 0.000005). Disease severity (MSSS) correlated with MST1 (ρ = 0.396, *P =* 0.00084) and APEH (ρ = 0.230, *P =* 0.05).

Linear regression analysis of serum protein levels and measures of disease disability/severity in all MS cases, adjusted for sex and age, revealed significant positive associations between PRDX6, MST1 and APEH levels and EDSS scores, while BST1 exhibited a negative association. After correction for multiple comparisons, only PRDX6 (*P*_FDR_ = 0.03) and MST1 (*P*_FDR_ = 0.00025) remained significant. Furthermore, MST1 (*P*_FDR_ = 0.012) and APEH (*P*_FDR_ = 0.024) were significantly associated with MSSS scores (**Table 7**).

**Table 7:** Summary of linear regression analyses examining association between serum protein levels and measures of disease disability/severity among all multiple sclerosis cases in the validation sample.

Protein	B	SE	P*	*P*_FDR_
Expanded disability status scale (EDSS)				
APEH	0.599	0.263	0.026	0.052
MST1	2.273	0.494	0.000021	**0.00025**
PRDX6	6554.42	2465.37	0.010	**0.03**
BST1	-1.235	0.534	0.024	0.057
Multiple sclerosis severity score (MSSS)				
APEH	0.509	0.181	0.006	**0.024**
MST1	1.124	0.356	0.002	**0.012**

P*, sex and age-adjusted *P*-value; *P*-_FDR_, FDR-adjusted *P*-value. Statistically significant values after FDR adjustment are indicated in bold.

**Figure 7:**
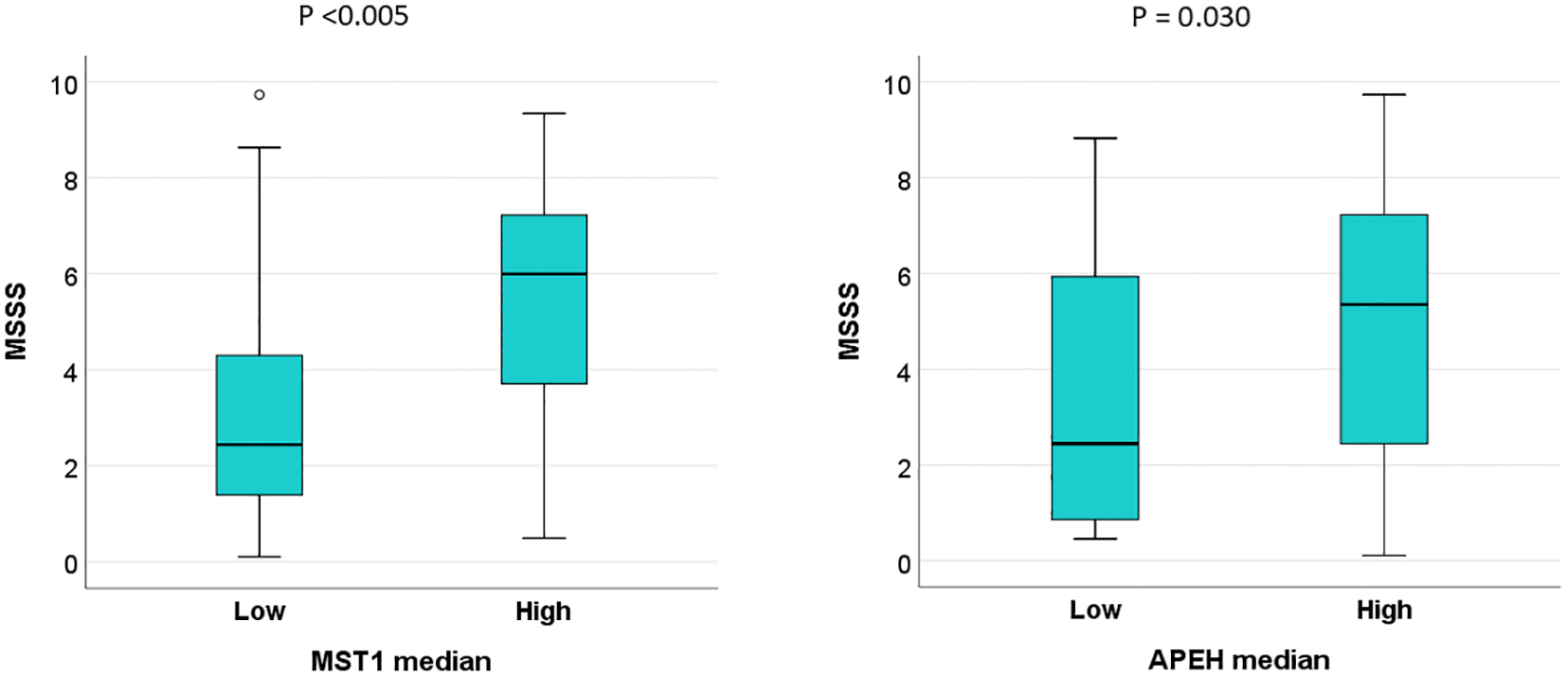
High serum levels of MST1 and APEH are associated with faster disease progression in MS patients in the validation sample. Multiple Sclerosis Severity Scores (MSSS) at the time of blood collection were analyzed in patients stratified into high and low abundance ratio (AR) groups for MST1 and APEH, using median cut-off values as the threshold.

To evaluate the potential prognostic implications of dysregulated protein levels in MS, MSSS at the time of blood collection was analyzed in patients stratified by high and low AR of these proteins, based on median cut-off values. MSSS scores were significantly higher in patients with elevated levels of MST1 and APEH compared to those with lower levels (**Figure 7**). No additional clinical correlations with protein levels were identified.

## 4 Discussion

Despite considerable research, our understanding of serum or plasma biomarkers associated with MS remains limited. Key challenges include the low abundance of many serum proteins and the uncertainty regarding how accurately they reflect the underlying mechanisms within the CNS during MS development. CSF remains the gold standard for proteomic biomarker discovery in MS, as it directly reflects ongoing pathological and inflammatory processes within the CNS. However, the accessibility and reproducibility of blood sampling have made serum proteomics an increasingly attractive alternative for identifying biomarkers of disease activity and progression in MS (17).

Our study followed the traditional, stepwise biomarker discovery paradigm. In the exploratory phase, we employed UHPLC-HRMS, incorporating high- and medium-abundance protein depletion and peptide fractionation to enhance detection sensitivity. This hypothesis-free analysis, conducted on a small but well-characterized sample of untreated MS patients, aimed to uncover candidate proteins with substantial dysregulation. As is standard in proteomics, we did not conduct formal power calculations for this initial phase, as the aim was not to draw definitive conclusions, but to generate a focused list of candidate biomarkers with large effect sizes for subsequent validation. This is a common and accepted approach in proteomics, where discovery phases are typically underpowered for small differences but optimized for detecting high-effect-size candidates for downstream validation.

Several methodological strengths distinguish our approach. First, by exclusively including untreated individuals, we eliminated potential confounding from disease-modifying therapies. Second, we applied stringent selection criteria—requiring a minimum two-fold change in protein abundance between groups—to focus on robust signals. Third, we validated our findings in an independent sample using ELISA, thereby enhancing reproducibility and minimizing the risk of false positives. A *post hoc* power analysis confirmed sufficient statistical power (0.86 at α = 0.05) to detect large effect sizes in this validation phase. Consistent inclusion and exclusion criteria across both samples further strengthen the internal validity of our results.

We fully acknowledge that, as a cross-sectional study, our design cannot capture longitudinal changes in biomarker levels over time or establish causal relationships with disease progression. However, longitudinal analysis was beyond the scope of this biomarker discovery phase, which was primarily designed to identify potential serum biomarkers in MS. Given the substantial time, funding, and multicenter collaboration required for such extended validation, longitudinal studies are warranted as a critical next step. Despite these limitations, our findings provide a valuable foundation for future research and contribute meaningfully to addressing the pressing need for serum biomarkers in MS.

In our exploratory analysis, we identified 13 serum proteins differentially expressed between MS patients and controls, with additional proteins distinguishing PPMS from ROMS. Of 15 total dysregulated proteins, 12 were analyzed in the validation phase by ELISA, due to sample limitations.

Some discrepancies between discovery and validation phases—such as those observed for PCSK9—are expected, as UHPLC-HRMS detects peptide fragments that may not correspond precisely with the intact proteins targeted by ELISA, especially in the presence of post-translational modifications. PCSK9, previously identified in other proteomic studies as being less abundant in the serum of MS patients compared to individuals with non-inflammatory neurological diseases (18) showed a lower AR in PPMS compared to RRMS during the exploratory phase; however, this difference was not significant in the validation phase.

Among the most robust findings in the validation phase were elevated serum levels of PRDX6, FCGR3A and APEH, particularly in PPMS patients, —and increased BST1 levels in ROMS patients. In contrast, CFHR5 and MST1 were significantly reduced in ROMS patients compared to controls, with MST1 also markedly lower in ROMS than in PPMS patients. Further subgroup analysis revealed that these reductions were predominantly driven by substantially decreased MST1 levels in RRMS patients, who also exhibited significantly lower levels than SPMS patients.

Although no significant differences in age or sex were observed between the overall MS group—including all clinical phenotypes—and the control group, phenotype-specific demographic variability may influence protein expression and should be taken into account in future studies. Larger, demographically stratified samples will be needed to adequately control for these potential confounders.

Importantly, serum levels of APEH and MST1 showed correlations with the MSSS, which, despite its limitations in capturing dynamic disease activity, provides a practical surrogate measure for relative disease severity in cross-sectional studies. Longitudinal investigations are needed to confirm the prognostic relevance of these associations.

Regarding the biological relevance of these findings, several proteins identified here have previously unrecognized roles in MS. For example, PRDX6, a multifunctional antioxidant enzyme with peroxidase, phospholipase A2, and acyl transferase activities (19, 20), was consistently elevated in MS, especially in PPMS patients.

Elevated serum PRDX6 levels have previously been reported in patients with MS and neuromyelitis optica spectrum disorder (NMOSD) compared to those with other neurological disorders, such as amyotrophic lateral sclerosis and spinocerebellar degeneration. While no significant correlations were found between serum PRDX6 levels and EDSS scores or disease duration in MS patients, a positive association was observed in NMOSD patients between serum PRDX6 levels and disease duration (21). Prior studies have linked increased PRDX6 expression in brain tissue from MS patients with pronounced astroglial proliferation (22) reduced BBB disruption (23) and neuroprotective effects (24). Moreover, increased PRDX6 expression in experimental autoimmune encephalomyelitis (EAE) mice led to reduced myelin loss, decreased MMP9 levels, and dampened microglial activation, thereby preserving BBB integrity and limiting immune cell infiltration (23).

Besides, administration of exogenous PRDX6 in EAE models improves BBB permeability primarily through its antioxidant (peroxidase) activity, which reduces ROS production derived from NADPH oxidases (NOX). Additionally, it modulates NOX enzymes by both enabling their activation and suppressing their gene expression, balancing oxidative stress at the BBB (25) thereby limiting peripheral immune cell infiltration and reducing neuroinflammation (23). Additionally, exogenous PRDX6 decreases pro-inflammatory cytokine production while promoting anti-inflammatory cytokines in macrophage cultures, helping to mitigate the chronic inflammation that drives MS progression (26). Thus, our finding of consistently elevated serum PRDX6 levels in MS patients along with its association with progression of the disease by means of EDSS, may reflect a compensatory, neuroprotective response, particularly in progressive forms of the disease. However, further validation in larger, longitudinal cohorts is essential to confirm PRDX6 diagnostic and therapeutic potential.

The FCGR3A, also known as CD16a, is the receptor for the invariable Fc fragment of IgG. Upon binding to clustered antigen-IgG complexes on cell surfaces, it undergoes activation, triggering the lysis of antibody-coated cells. Then, it is released as a soluble form through proteolytic cleavage (27). Although data on soluble FCGR3A (sFCGR3A) in MS are sparse,—with one non-validated proteomic study in Chinese MS patients reporting decreased plasma levels of sFCGR3A in pwMS compared to HC (28)—the transmembrane form of FCGR3A is known to be highly expressed on nonclassical monocytes/macrophages (CD14^+^CD16^++^), CD16^+^CD56^dim^ NK, and γδ T cells (29) as well as on microglia, oligodendrocyte precursor cells and immature oligodendrocytes (30). In our study, patients with PPMS showed a higher abundance of sFCGR3A compared to HC. While the exact source of sFCGR3A in these patients remains unclear, it is likely generated through proteolytic cleavage from the surface of activated nonclassical monocytes or CD16^+^CD56^dim^ NK cells. Supporting this hypothesis, increased frequencies of nonclassical monocytes expressing surface FCGR3A have been reported in pwMS when compared to controls (31–33). Upon activation, these monocytes adopt a pro-inflammatory phenotype, exhibit strong T cell-activating capacity, efficiently migrate across the BBB, and may also contribute to BBB disruption (34). Studies on membrane FCGR3A expression in NK cells in MS have yielded mixed results: some report decreased cytotoxic activity of CD16^+^ NK cells in RRMS patients, correlating with disease activity (35) while others found no significant differences in the percentages of NK cells expressing or lacking CD16 between untreated RRMS patients and HC (36), or even higher percentages of these NK cells in PPMS and SPMS patients (37). In this context, our results suggest that increased sFCGR3A may reflect immune cell activation and BBB disruption, potentially serving as a marker of inflammatory burden in progressive MS.

The third protein with a high AR in the serum of all clinical forms of pwMS compared to HC was APEH. Although no previous studies have directly linked this protein to MS, its association with the MSSS suggest a potential role in the pathophysiology of MS, particularly during the progressive phase. APEH is an enzyme that degrades bacterial and mitochondrial proteins by hydrolysis, generating acetylated peptides that can drive inflammation (38) and its activity may be inhibited under oxidative stress conditions (39) While the link between increased APEH expression and disease progression remains unclear, its dual roles in immune activation and redox balance, position APEH as a promising biomarker for both inflammation and oxidative damage in MS. These findings warrant further mechanistic studies to determine whether increased serum APEH levels in pwMS reflect the release of proinflammatory acetylated peptides and to clarify the role of APEH in lipid peroxidation and its potential as a therapeutic target.

The last protein with a markedly higher abundance in pwMS compared to HC in our analysis was BST1. This protein is expressed on myeloid and neural cells (40), and facilitates leukocyte adhesion and transmigration across the BBB, promoting neuroinflammation (41). Its elevated levels in ROMS compared to HC may reflect the activation of innate immune responses that trigger neuroinflammation. This is particularly relevant in the context of MS, as myeloid cells, are key players in the inflammatory processes that drive demyelination and neurodegeneration. BST1 has been implicated in other autoimmune conditions such as rheumatoid arthritis and certain malignancies (41, 42) suggesting shared inflammatory pathways. The parallels between these conditions and MS, particularly in terms of chronic inflammation and tissue damage, highlight the potential of BST1 as a biomarker for MS, especially in its relapsing-remitting form.

Conversely, CFHR5, a regulator of the complement system via C3b binding (43), was significantly reduced in ROMS patients compared to HC, suggesting impaired regulation of innate immune responses. This observation hints at a possible role of CFHR5 in MS pathophysiology, given its involvement in other autoimmune diseases where both complement system deficiencies and overactivation contribute to disease development and progression (44), such as systemic lupus erythematosus (SLE) (45). The reduced levels of CFHR5 in ROMS patients may indicate a dysregulation in the innate immune system, potentially exacerbating the inflammatory responses characteristic of relapsing MS.

Finally, MST1 demonstrated a distinctive profile, with significantly reduced serum levels in RRMS patients compared to HC and the other MS clinical forms. The mechanism through which MST1 regulates immune responses remains unclear. However, MST1 has been implicated in the modulation of innate immune system (46), as well as in the regulation of naïve T cell proliferation, and its deficiency has been reported to impair T_reg_ development and functions in mice (47). It has also been reported as a proapoptotic molecule that increases ROS production and is able to mediate H_2_O_2_-induced cell death in cultured mouse astrocytes (48), and IFN-g-induced apoptosis in rat microglial cells (49).

Regarding MS, a previous study has reported elevated MST1 levels in T-cell derived extracellular vesicles (EVs) from RRMS relative to HC, identifying it as a white matter injury-related protein (50). These findings align with our results, as proteins encapsulated within EVs are not detected by ELISA without prior vesicle lysis. This may also explain why, in our proteomic analysis involving sample sonication and precipitation and thus, EVs lysis, MST1 levels in RRMS patients were 2.547 times higher than those in HC. We cannot determine whether the marked decrease in serum MST1 levels observed in RRMS patients compared to HC and other clinical forms contributes to immune dysregulation—primarily through T_reg_ dysfunction, promoting a shift toward proinflammatory T cells that may drive neuroinflammation—or simply reflects its high concentration in T-cell-derived EVs, or both.

While some of the proteins identified have also been reported in other neuroinflammatory and autoimmune diseases which may limit their specificity to MS, they remain biologically relevant and may be reflecting shared inflammatory pathways. For instance, elevated serum PRDX6 levels have been reported in both MS and NMOSD compared to other inflammatory neurological diseases (21) and in SLE, urinary PRDX6 levels correlate with disease activity (51).

Regarding FCGR3A, it has been functionally implicated in rheumatoid arthritis (RA), where circulating and synovial immune complexes have been shown to activate *FCGR3A*, supporting its pathogenic role in immune complex-driven inflammation (52). In SLE, certain *FCGR3A* polymorphisms have been associated with increased disease risk (53); however, attempts to validate its increased expression by qPCR in SLE were unsuccessful (54), highlighting the complexity of transcriptomic-proteomic correlations. Similarly, CFHR5, has been found elevated in SLE (45), lupus nephritis and ankylosing spondylitis, a chronic inflammatory rheumatic disease, where it holds potential diagnostic and prognostic value (55, 56). This overlap underscores shared immunopathogenic mechanisms across autoimmune diseases. Nevertheless, the distinct expression patterns observed across MS phenotypes suggest potential utility for these markers in disease stratification and monitoring, even if they lack disease exclusivity. Future studies should include comparisons with other autoimmune or neuroinflammatory diseases to further delineate disease-specific signatures and improve the diagnostic utility of these biomarkers.

Finally, it is important to consider that serum protein profiles are highly dynamic, influenced by environmental and physiological changes (6, 11), and inherently lack tissue or cell-type specificity (17). Thus, stringent phenotypic characterization, as applied in this study, is critical to ensure meaningful interpretation of results.

## 5 Conclusion

In summary, our findings highlight a panel of serum proteins with differential expression across MS clinical phenotypes, many of which are involved in pathways central to MS pathophysiology—including oxidative stress, immune regulation, and BBB integrity. While most of the identified proteins appear to promote inflammation or oxidative damage, PRDX6 may exert neuroprotective effects. These proteins represent promising candidates for future validation studies and could aid in stratifying patients by disease subtype or progression risk. However, these findings should be interpreted in light of certain limitations. As a cross-sectional study, our design does not permit evaluation of temporal changes in biomarker levels or their dynamic association with disease progression. Moreover, the generalizability of our results may be constrained by the demographic composition of our sample, which consisted predominantly of individuals of Southern European descent. To strengthen the clinical relevance and applicability of these biomarkers, future studies should prioritize longitudinal designs and include larger, multiethnic, and multicenter cohorts before translation to clinical practice. Additionally, incorporating patients with a broader range of neurological disorders as controls will be essential to assess the specificity and diagnostic value of the identified proteomic signatures.

## Data Availability

The proteomic data from the exploratory phase of this study, titled Proteomic data in MS-Málaga, are publicly available in the Mendeley Data repository. The dataset can be accessed via the following DOI: https://doi.org/10.17632/3dkp55dfr9.1.
