# Supplementary material for: Discovery and early validation of serum protein signatures in untreated multiple sclerosis patients: identification of candidate biomarkers for diagnosis and stratification
Source: Front Immunol. 2025 Aug 21;16:1579045. doi: 10.3389/fimmu.2025.1579045 (PMC12408279; doi:10.3389/fimmu.2025.1579045)
Supplement: Supplementary file 1 [file Table1.pdf]

Supplementary Table 1. Detection ranges, analytical sensitivities, lot numbers, intra- and inter-assay coefficients of variation (CV) of the ELISA kits used in the validation phase.

Protein	Detection ranges	Sensitivity	Batch number	intra-assay CV	inter-assay CV
ACTN1	0.31 - 20 ng/ml	0.115 ng/ml	L210322284	<10%	<12%
MST1	156 - 10000 pg/ml	<10 pg/ml	4651739330	<8%	<10%
APEH	0.156 - 10 ng/ml	0.094 ng/ml	H1573G038E	<8%	<10%
CFHR2	0.313 - 20 ng/ml	0.188 ng/ml	H4862G120, H4862G038E	<8%	<10%
ELANE	0.313 - 20 ng/ml	0.188 ng/ml	H0750G038E	<8%	<10%
BST1	0.156 - 10 ng/ml	0.094 ng/ml	H2142G120, H2142G038E	<8%	<10%
CFHR5	31.25 - 2000 pg/ml	14.8 pg/ml	L210621439	<10%	<12%
FCGR3A	31.2 - 2000 pg/ml	13.5 pg/ml	L211126630	<10%	<12%
PGAM1	0.31 - 20 ng/ml	0.125 ng/ml	L210322253	<10%	<12%
PRDX6	15.625 - 1000 pg/ml	9.375 pg/ml	H1911G120, H1911G038E	<8%	<10%
PCSK9	0.030 - 0.219 ng/ml	0.096 ng/ml	P266024, P277732	<7%	<6%
S100A6	78 - 5000 pg/ml	30 pg/ml	L210322253	<10%	<12%
